# The Causes, Nature, Diagnosis, and Treatment of Acute Hydrocephalus

**Published:** 1843-04-01

**Authors:** 


### The Causes, Nature, Diagnosis, and Treatment of Acute Hydrocephalus. A Prize Essay. By *James Risdon Bennett*, M.D. Octavo, pp. 248. Highley, 1843.

We always open a new work upon consumption, cancer, water in the head, or other deadly disease, with a melancholy interest, anxious, yet scarcely expecting, to discover some new means of compassing our great antagonist, and forcing him from these his strongholds; and we usually close it with vexation at finding that the number of our implements of warfare is not augmented, although the system of tactics may be varied, while means are too often held forth as panaceæ with a confidence and appeal to facts which is startling and would prove convincing, had not a bitter experience engendered an incredulity, only to be removed by a rigorous demonstration, repeated under every variety of circumstances. The present work professes no such discovery, and, indeed, one of its chief claims to the very favorable notice we feel called to bestow upon it, is derived from the spirit of careful discrimination, and the absence of hasty generalization which pervade it throughout.

Dr. Bennett, having become a long time since aware that very vague and contradictory opinions prevailed respecting this important disease, resolved, when the London Medical Society proposed its consideration as the subject for the Fothergillian Gold Medal for 1842, to submit his own and other's experience to an impartial examination, with the hope of reconciling some of the inconsistences and illuminating some of the obscurities. And in this we think he has succeeded.

The work is divided into seven chapters, each of which we will now submit to analysis.

### Chap. I.—Varieties of the Disease and Order of the Symptoms.

The author truly observes that much of the obscurity in this affection has arisen from the adoption of the same name for the designation of very different conditions of disease. After enumerating, in a very distinct manner, the various *premonitory symptoms*, both those referable to the condition of the digestive organs, and those arising more directly from the altered condition of the nervous centres, he proceeds as follows:

"For my own part, I have been led to consider the very marked change from the natural aspect and expression of countenance, the listless, vacant expression of eye with the dark line beneath, and a constipated state of the bowels, with evidence of deficient action of the liver, when associated with any of the above symptoms more directly referable to the brain, as calling for very great watchfulness on the part of the medical attendant, and as justifying serious apprehensions of the invasion of hydrocephalus; and I entertain a strong conviction that judicious treatment in such circumstances often prevents the accession of dangerous cerebral disease. * * * * *

"I cannot avoid urging on the attention of the reader the great frequency of derangement of the liver and an inactive state of the bowels as preludes to hydrocephalus, and the manifest propriety, in suspicious circumstances, of not merely adopting occasional and temporary means for the relief of these symptoms, but of giving them the most serious and continued attention." 11.

Dr. Bennett describes four varieties of genuine hydrocephalus.

"*Of the First or Gradual Form.*—The nervous of Hopfengärtner, the nervous and ataxic of Breschet, the ataxic of Guersent, and the third and fourth forms of inflammatory disease within the head as described by Abercrombie. This, although the more chronic, is the more frequent form under which what is called acute hydrocephalus is met with in practice; and it is on many accounts the most important, though the least understood by the older nosologists."

The *premonitory symptoms* may exist for weeks or months before the disease becomes fully developed, but a post-mortem examination proves to us, by reason of the extent of the changes which have taken place, that these symptoms must sometimes be considered rather as attendant upon actual disease, existing in a chronic degree, than as merely premonitory. The early symptoms are especially liable to be overlooked or misinterpreted, if the child be recovering from some previous debilitating disorder, or if frequently ailing from a sickly and delicate constitution. They are frequently attributed to mere gastric fever or worms, and, in some cases, bear no distinct reference to the head until near the termination of the disease. When the *headache* appears it is frequently confined to one part of the head, and is usually marked by sudden paroxysms, producing shrieking. Sometimes the child is continually moaning, keeping its hand to the head, and uttering such plaintive and peculiar cries, that Coindet considers the hydrocephalic cry to be in some measure diagnostic. When spontaneous *vomiting* occurs the case becomes more clear, and both this symptom and the headache are encreased by motion and the erect posture. They are usually preceded by chilling and flushing of the face, for a day or two before, but, as a general rule, the child's face is pale, unless exertion produce a transient flush. The *pulse* is usually frequent, and often somewhat sharp, but very excitable and variable. Dr. Bennett is not disposed to deny that it may occasionally be found natural or slow, at this early period, although the latter characteristic is more frequently found towards the termination of the disease. The temperature of the *skin* is seldom much encreased, and may even be diminished. It is harsh and dry, especially about the nose and lips, as noticed by Gölis. Tongue furred, breath very offensive, bowels constipated or irregular, urine scanty, turbid, and variable, but furnishing no characteristic appearance.

The various symptoms of what is termed the *first stage* of the disease, denoted by the tossing the head upon the pillow, sensitiveness to light and sound, knitting of the brow, contraction of the pupil, screaming, &c. may last for ten, twelve, or fourteen days, when they pass into the somnolent and convulsive stage. What is termed the *third* stage seems merely an aggravation of the second, while, in many cases, it is difficult to make out the division of the disease into stages at all, owing to the endless variety in the order in which the symptoms may occur.

"It is important, however, to distinguish between the symptoms which may be fairly attributed to excitement of the brain, to exalted action; and those which indicate an oppressed condition of the sensorium and torpor, or obliteration of its functions; or, in other words, those phenomena which indicate more or less complete coma. For, these latter symptoms, in all cases, I believe, must, to a certain degree, be attributed to the *effects* of previous morbid action, whatever may be its nature." 21.

2. The *Second* form is termed the *Insidious,* and by its insidiousness chiefly differs from that already mentioned. Dr. Abercrombie has particularly described this variety, and the author agrees with him in assigning a little above puberty as the age at which it most frequently occurs, although, it is sometimes found between seven and ten. It commences as a common febrile attack, and the patient may suffer from remissions and aggravations for some weeks before the headache becomes very constant, when, although not severe, it is now accompanied with a sense of oppression, and unwillingness to be disturbed.

"These symptoms, on enquiry, it is difficult to account for: the pulse at the same time being almost natural, having lost the frequency it may have had previously, and the tongue being clean, and the appetite improving. The symptoms of general disturbance do not, however, accord with the permanence and amount of headache. In this way several days more, or, I should say, even a week or two may elapse, when the headache increases with an evident tendency to stupor, and with a falling of the pulse, and then the disease becomes fully developed. In infants the symptoms in the early stage are sometimes so trifling that no alarm is taken till the child is in a state of stupor, which soon lapses into coma, generally attended by squinting. In some of these cases Dr. Abercrombie observes, there is still less appearance of disease of the head, and there is not the slightest complaint of headache through the whole course of the complaint." 23.

3. *The Acute, Febrile, or Inflammatory Form.*—In this, the symptoms may be subacute, when they much resemble the first form, or they may, in other cases, be hyper-acute, occurring without any premonitory symptoms. Dr. Cheyne's admirable observations are quoted by the author.

4. *Consecutive or Secondary Hydrocephalus* supervenes on the subsidence of some other disease. This is a very acute form, and if not promptly met rapidly proves fatal.

"I would not class under this head cases that occur during, or subsequent to dentition, nor those which attend the termination of, or succeed to, chronic affections, particularly of a scrofulous character. Most of these, I believe, are as much entitled to the denomination of idiopathic as any form of the disease. It is chiefly on the subsidence of acute febrile disorders, and particularly after scarlatina, that the cases occur to which the form *secondary* appears to me strictly applicable. If we were here considering the disease as occurring to adults as well as to children, to the cases I have mentioned should be added those which supervene on acute disease of the liver and kidney. Keeping in view this limitation of the term secondary hydrocephalus, it will be found generally to present much of the violent character of the third variety. The face, however, is generally pale, the pulse quick, the secretion of urine scanty; and particularly after scarlatina, there generally is, or has been, more or less anasarca. Headache, though sometimes not much complained of, generally precedes all the other cephalic symptoms, and is often almost the only one indicative of the approach of the disease. * * * It is to this variety of the disease that the term water-stroke is most applicable, and I quite agree with Dr. Copland, that it hardly ever occurs in a strictly idiopathic form." 27.

*Pseudo-Hydrocephalus.*—Under this head Dr. Bennett reviews the various forms of head-affection likely to be confounded with genuine hydrocephalus.

(A.) *The Hydrocephaloid Disease.*—Dr. Bennett contents himself with referring to the valuable essays of Abercrombie, Hall, and Gooch, agreeing with the latter, that causes of prior exhaustion, supposed by Hall always to be present, are not so necessarily though usually. "Such changes in the circulation, are, no doubt, the most frequent and important causes of the functional derangement, of the altered vital properties of the brain; but alterations in the quality of the blood, in the nutrition of the brain, may equally induce the state of things described by Dr. Hall and his fellow observers. Hence, defective nutrition of the body, and an imperfect supply of the other vital stimuli, particularly of light and air, may, by first inducing a state of irritation, eventually induce congestion, or the symptoms simulating hydrocephalus; and I think I shall be borne out in the statement, that it is in debilitated, weakly children, and chiefly those of the poor, that the hydrocephaloid disease is seen, except where it manifestly succeeds direct exhaustion." He agrees with Evanson and Maunsell that *squinting* is very often present in this affection, as it often is in slight and temporary derangements of the brain in delicate children, who are suffering from derangements of the biliary secretions, and gives rise to unnecessary alarm.

(B.) *Erethism, or Irritation of the Brain in Infants.*—This is a condition of the brain, described by Dr. Nickoll, "in which inordinate effects arise from ordinary impressions on different parts of the nervous system," and which often resembles the first stage of hydrocephalus. It occurs especially in scrofulous children, and is frequently produced by the irritation arising from painful dentition, worms, disordered secretions, burns, ulcers, surgical operations, &c. As it is accompanied by encreased determination to the head, and unpreceded by causes of exhaustion, it is more likely to be mistaken for hydrocephalus than is the hydrocephaloid disease itself. If it be treated by antiphlogistic means, exhaustion and fatal effusion will supervene, while, if the causes of irritation be removed, the sources of excitement excluded, and mild sedatives administered, recovery soon takes place.

(C.) Children are subject to a state of the brain which may be termed *Torpor*, manifested by a very unexcitable state of system, and coming on slowly in the course of a prolonged and debilitating illness. Dr. Bennett has found it connected with a vitiated state of body, requiring a prolonged course of tonics, especially iron. It occurs under the same circumstances as erethism, and the difference of effect must result from the great difference of nervous constitution in the individuals.

### CHAP. II.—STATISTICS OF HYDROCEPHALUS.

Dr. Bennett but expresses the general feeling of the profession when he acknowledges the great obligations due to Mr. Farr, for his labours in the important field of medical statistics. It is from these chiefly that the present chapter is compiled.

1. *Frequency of the Disease.**—In the half year, to which the first Report of the Registrar General relates, there died 78,487 under 15 years of age, of which 3,570 were from hydrocephalus, or more than 4½ per cent. In the year ending June 1839 there were 155,406 deaths under 15, and of these 7,672 were from hydrocephalus, or nearly 5 per cent. According to the last registration, ending June 1840, there were 169,675 deaths under 15, and 7,769 were from hydrocephalus.

2. *Influence of Season.*—Great difference of opinion prevails upon this point, for, while Guersent states he never met with a case in the burning heats of Summer, other authors state that it is more frequent at that season than in Winter. Extracts from the Registrar's Tables are adduced by Dr. Bennett, as proving the great predominance of the number of deaths from this cause in *Winter,* compared to Summer and Autumn. He believes, also, from the fact of cases of hydrocephalus seldom occurring singly, but becoming frequent at certain periods, and then disappearing for a while, that the meteorological condition of the atmosphere must have considerable influence.

3. *Influence of Climate.*—This section is almost a misnomer, seeing that it merely alludes to the comparative prevalence of the disease in large cities and rural districts of the same climate. The conclusions at which the author has arrived, from the examination of various tables, are thus expressed.

"Whence it appears that the deaths from hydrocephalus in the metropolis are nearly treble those of the south-western counties, and more than treble those of the counties of Norfolk, &c., and nearly one-third more than those of the twenty-four towns. Now the difference, it must be observed, in the gross mortality of the metropolis and the agricultural districts is by no means so great as this, being only about two-fifths more. The difference of mortality in the whole class of nervous diseases in towns and in the country is greater than in most other classes, and considerably greater than in diseases of the respiratory system, which, of course, includes pulmonary consumption." 52.

Dr. Brigham states that, while in the last thirty years the population of New York has only quadrupled, the deaths from inflammation and dropsy of the brain have increased twelve-fold. In Boston "the population in 1790 was 18,038 with 201 square yards to each inhabitant; and in 1837, 80,325 with only 49 square yards to each person. The increased density, therefore was as 5 to 1; and, while the mortality per 1000 from apoplexy and convulsions remained nearly the same, that from hydrocephalus was more than trebled."

4. *Influence of Sex.*—Most writers state the disease to be most frequent in females, but the tables here quoted prove quite the contrary; and this becomes the more observable when a comparison is instituted with respect

* Fully as we appreciate the benefits derivable to the science of medicine from the Death's Registration Act, we must consider it as but an imperfect measure, until means are adopted for ensuring a more accurate statement of the cause of death being delivered to the assistant registrars, than takes place at present. In the great majority of instances a medical man has been in attendance prior to the death, and a certificate from him should always be required.

to other diseases. Thus, in the report for 1838, (*e. g.*) 4,242 males and but 3,430 females are registered, while in hooping-cough there were 4,036 deaths of males, and 5,071 females; in consumption, 27,935 males, and 31,090 females. "So that, while consumption appears to be 8 per cent. more fatal to females than males, hydrocephalus is 20 per cent. more fatal to males than to females. Diseases of the nervous system in general are 23 per cent. more fatal to males than to females, and the chief difference appears to arise from the diseases affecting children, of which hydrocephalus is a striking illustration."

5. *Influence of Age.*—Of 265 cases, 182 occurred under the age of 7, while only in 46 did it exceed 10, and the number of cases under one year is very small. The disposition to the disease is greatest between 2 and 7.

6. *Duration.*—Of 117 cases collected by Green, 80, or nearly two-thirds, terminated within the first fortnight, and only one-eighteenth exceeded three weeks. Of 15 related by Schweninger, the mean duration was 17 days. Of 28 cases by Gölis, 18 terminated within the first fortnight, and only two exceeded 20 days. Dr. Bennett's experience coincides with the above, and he states the disease seldom extends beyond three weeks.

### CHAP. III.—MORBID ANATOMY.

Almost every author of note has related cases (few in number it is true) in which effusion of fluid has been the only discernible result of acute hydrocephalus—it being totally unaccompanied by marks of inflammatory action.

Although after acute hydrocephalus the whole substance of the brain is usually found softer, yet it sometimes retains its healthy firmness. A very frequent change is a greater or less degree of *local softening of the white central portions*, usually accompanied by effusion, but at other times existing without any. A coincident inequality of thickness, and deep blue colour, of the cranial bones are often observed. This central softening is the most frequent of all changes in hydrocephalus. The serum is usually limpid and quite clear. When, however, the softening is very great, the examination delayed, or clumsily performed, the fluid often becomes turbid, owing to the commixture of the previously clear fluid with portions of the softened cerebral matter; and this turbidity is erroneously supposed to arise from the presence of coagulable lymph, pus, or some of the products of inflammation.

Dr. Bennett enters into some detail to prove from analogy the correctness of the suggestion of Andral, that this central softening is not necessarily inflammatory, but "a specific alteration of nutrition which may supervene under the influence of morbid conditions widely different from each other." He thus concludes—

"Finally, I would observe, and it should not be forgotten, that, as the softening of which we are speaking is often, or rather generally unattended by any of the physical characters of inflammation, so it is often seen in cases that have not manifested during life any symptoms of inflammation, and that it frequently occurs in circumstances opposed to the supposition of inflammation being its cause. In hydrocephalus, it is true, it is often seen in those cases in which the symptoms have been of the most acute character, and in which, if they have been really attributable to inflammation, it must be considered as of an acute, and not a chronic character, and yet we find none of the ordinary effects of acute inflammation that has lasted for many days. But even in the simple cases followed by effusion only, the symptoms during life sometimes so closely resemble those attending inflammation within the head, that they are with difficulty distinguished; yet we know that many of these cases are the result of mere irritation, or of direct exhaustion of the system. To the hygrometric properties of the brain, already referred to, it should also be remembered, we must refer many of the cases of central softening found after death; for it has been fully proved, by Dr. Paterson, that the parts nearest to the cerebro-spinal fluid are the first to manifest pseudo-morbid softening after death: and there can be very little doubt that many of the reported cases of softening from disease have been nothing more than instances of this. The period after death at which the examination takes place is not always mentioned, nor taken into consideration as it ought to be." 90.

The quantity of *fluid* found in hydrocephalus is very variable, and in no wise proportionate to the severity of the symptoms; and it is now generally admitted that its quantity, and its existence at all, are of comparatively little consequence. The normal quantity also which should be found in the ventricles may become increased, when any circumstance, offering impediment to the cerebral circulation, has existed prior to death. "As a general rule, the more acute the disease the less water will be found, and *vice versa*." There is generally more also found in these cases of affection of the central parts of the brain than where the membranes are inflamed. Dr. Bennett's experience agrees with that of Nasse, "that, *cæteris paribus*, the younger the child the higher the average quantity; but that the amount seldom exceeds ℥vj. in cases having any claim to the title of acute, and is not usually above ℥iij or ℥iv." Guillot has proved by his experiments that the brain can take up by imbibition a quantity of water equal to its own weight, and thus we sometimes find the ventricles empty, but enlarged in size, from former distention. Effusion beneath the arachnoid does not usually co-exist with the ventricular, which is attendant upon central softening, and this membrane is often found preternaturally dry.

In a large proportion of cases of acute hydrocephalus, more than one-half, meningitis is present, and contrary to what occurs in adults, the base is the seat of it far more frequently that the convexity. When it is confined to the convexity the ventricles frequently contain no fluid, while it is seldom we find meningitis of the base without effusion into these cavities.

The frequent association of meningitis with more or less inflammation of the substance of the brain, and the effusion of fluid into the ventricles, has led many authors to describe hydrocephalus as a mere cephalitis of children. But, if we omit the cases of central softening, already alluded to, we shall find, in this disease, marked inflammation of the substance of the brain in but a small number of cases, although its cortical surface is not unfrequently affected from extension of the meningeal inflammation.

Bloody points are often unusually frequent within its substance, whether meningitis is present or not. The consistence of the brain is sometimes less, sometimes greater than ordinary. In connexion with the inflamed condition of the brain, there may be the same central softening, with a limpid condition of the fluid of the ventricle, and the absence of all marks of an inflamed state of the membranes lining this cavity, already adverted to.

But there is another class of cases in which there is "undoubted evidence of inflammatory action *within* the *ventricles, e. g.* effusion of fibrine or pus, and changes in the serous membranes lining these cavities, evidently resulting from inflammation. These cases are, for the most part, associated with inflammation of membranes of the base." The turbid appearance of the fluid, in these comparatively very rare cases, forms a great contrast with its clear and transparent state in so many other cases of hydrocephalus.

Dr. Bennett next describes the various indications of *scrofulous action*, found after death, in conjunction with the appearances proper to hydrocephalus. In some subjects is found effused by the membranes a yellowish gelatinous substance, frequently containing caseous deposit, and intermediate in texture between fibrine and tuberculous matter. It is often seen at the base of the brain, but here, simple serum effused into the meshes of the pia-mater, until cut into, often presents the same appearance. In other cases, decided tuberculous matter is deposited in layers or patches, varying in size and consistence, or, again, as solid masses imbedded in the substance of the brain. Again, granules or miliary tubercles may be observed, singly or in groups, situated on the pia mater. The modern French pathologists, considering these as scrofulous, and associating with them the symptoms of hydrocephalus, have named that disease tubercular meningitis. Dr. Green has taken great pains in making these opinions known in this country; but, with us, they have received but little confirmation, so that, as Dr. Bennett observes, this form of the disease is probably less frequent here than with our neighbours, and that it is little else than a mere assumption to regard these granules as of a scrofulous nature. Still, the very general admission of the connexion between scrofula and hydrocephalus, renders it a matter of surprise and regret that the other organs of the body have not, in cases of hydrocephalus, been more frequently examined in reference to this point. Of 30 cases examined by Gerhard, 29 presented tubercles in some important organ, as did 18 out of 20 cases examined by Green. Of 20 cases reported by Schweninger, the lungs were affected in 15, and the bronchial glands in 17. It is only quite in recent times that these latter organs have been submitted to examination at all. The alterations in many of the abdominal viscera, even when not consisting of positive tubercle, are, usually, as reported by various authors, of a scrofulous character.

### CHAP. IV.—ÆTIOLOGY.

Of the *Predisposing Causes* the condition of the brain in infancy is a principal. From the first to the seventh or eighth year its activity of growth and function is continually augmenting, as is its density of structure.

The influence of various remedies also shew these to be peculiarities at this age. Thus, opium requires the greatest care in its administration, while the system is notoriously insensible to the specific action of mercury, which nevertheless acts admirably as a purgative. Mere excess of nutrition of the brain may predispose; and, where there is great functional activity, and susceptibility, the circulation through the organ may easily become disturbed by irritation at the peripheral extremities of the nerves, and, that it often is so, the effects of difficult dentition, the presence of worms, or a disordered state of the secretions of the canal, sufficiently prove; and the repetition of such irritation continually encreases the predisposition to cerebral disease. The danger of encouraging the intellectual exertions of precocious children is known to every medical man. Operating upon this state of brain, the changed constitution of the blood, from a depraved condition of the assimilatory organs, must be taken into consideration. Dr. Bennett attaches great importance to the influence exerted by the disordered state of the biliary secretions, so frequent in young children. But it is to *scrofula* as a predisposing cause that he especially directs attention; believing, that although the connexion between the diseases has been pointed out by some of our best writers, yet "this very important fact is either not generally admitted by the profession, in this country, or has not received the consideration which it deserves."

*Exciting Causes.*—These are often obscure. Among them may especially be mentioned external violence, as from falls or blows; but the influence of these is often lost sight of, by reason of the very long period that may elapse before the effects manifest themselves. Violent mental emotion, as from severe fright, has often produced the disease. The influence of other diseases is considerable; thus, fatal effusion may attend pertussis, the gastro-enterite of weaning, and the phthisis of children. Suppression of evacuations and of discharges, especially when existing about the head and ears, and the disappearance of the exanthemata may induce it. The influence of cold is also very considerable, and this is very important to remark; for, a practice of keeping young children's heads inordinately cold, has, of late, been substituted for the opposite error of maintaining them far too hot.

"The evidence throughout all climates is uniform in regard to the influence of cold in favouring cerebral congestion, the frequency of which increases with the diminution of temperature. In Holland, Rome, Turin, Paris, and in this country, taking the average of a number of years, it has been clearly ascertained, that, in respect to the frequency of cerebral congestions, Winter is the season that stands first in order. Sudden transitions from one extreme to another have also been found to be attended by an increased frequency of the same disease. The conclusions derived from the tables I have already given, shew that hydrocephalus also is more frequent in the colder months than in the hotter." 13.

Many wretched mothers induce this disease in their children by administering to them narcotic drugs and spirituous liquors.

### CHAP. V.—PATHOGENY.

The author comments upon the extraordinary statement of Gölis, that he had found the organic products of inflammatory action in 180 cases. As this is so contrary to the experience of every other observer, he considers he must have often mistaken the infiltration of the arachnoid, and the turbidity (accidental) of the fluid of the ventricle for these. Localities, however, seem to have much effect upon the nature of the disease; for, while Dr. Clark's practice principally recognized a highly inflammatory description of disease, the experience of the Parisian hospitals seems to relate chiefly to a scrofulous variety. The conclusions the author arrives at are as follow.

"1. That in many instances the disease consists simply in *inflammation of the brain and its membranes;* the symptoms and the post-mortem appearances varying accordingly as the inflammatory action is seated primarily in the substance of the brain, or in the meninges, and according as it is more acute or chronic; and that in some of the most acute forms, rapidly terminating in death, little or no effusion may be found.

"2. That in by far the largest class of cases *the disease is essentially the result of scrofulous action,* and may, or may not, be attended by the signs of inflammation; that the most characteristic lesions in these cases are softening of the central parts of the brain and the effusion of serum; but that meningitis, chiefly of the base, is a very frequent secondary lesion, and is usually of a manifestly strumous character; and that, therefore, in this, the largest and most fatal class, acute hydrocephalus is but a modification of scrofulous disease."

He explains himself more fully upon this point in another part.

"The conclusions, therefore, to which I have been led, are;—That vital changes in the brain, chiefly in the central white parts, of the character probably of tubercular degeneration, are the primary causes of the simple forms of hydrocephalus: and that softening, effusion into the ventricles, and meningitis, are all consequences of antecedent alterations of nutrition:—That in some cases derangement and suspension of the cerebral functions may be induced, and lead to a fatal termination, either previously or subsequently to the occurrence of effusion, and, a *fortiori,* before any inflammatory action has been set up:—That in other cases it is the secondary inflammation which is the immediate cause of death, and that this inflammation generally partakes of the original scrofulous character of the disease, as manifested by the various forms of tubercular deposition by which it is attended:—That in other cases the scrofulous nature of the disease is intimated by the presence of distinct tubercular masses in various parts of the brain, which lead eventually to a fatal termination by the irritation and inflammation they excite." 149.

To continue the conclusions—

"3. That there are cases, from their symptoms, hardly to be distinguished from the last class, in which effusion into the ventricles is the only morbid appearance met with after death; and that in these instances the essence of the disease appears to consist in some alteration in the condition of the nervous matter, probably allied to irritation, and that they may, therefore, be said to constitute a purely *nervous variety.*

"4. That there is a class of cases distinct from the above, but closely allied to them, which may be generally traced to some source of exhaustion, either direct or indirect, in which the post-mortem appearances are generally indistinct and of a trifling kind, consisting, for the most part, of some degree of congestion of the large vessels, and a little effusion of serum; and that, in some of these cases, the effusion has probably resulted from injudicious treatment, had recourse to with a view to cure an imaginary inflammation; these being the cases described by Dr. Hall and others under the designation of '*Hydrencephaloid Disease.*'" 157.

### Chap. VI.—Diagnosis.

We regret that our space will not admit of our transcribing some excellent remarks upon the importance, not only of forming a correct diagnosis, as to whether cerebral disease exists at all, but, also, as to what particular form of it may be present. There are numerous other important observations, however, that we must not pass over.

In its *sthenic* form, the disease is usually recognised by the activity of its symptoms. There is a considerable similarity to the precursory symptoms of small-pox, but the *rigors*, which are usually so distinctly present in the latter, are not so in hydrocephalus.

With respect to the variety consecutive on scarlatina, the author makes the following remarks.

"In that form of hydrocephalus supervening on scarlatina, (and more especially on measles,) the diagnosis is not usually attended by much difficulty, though, in some instances, its approach may be very insidious. Any degree of headache associated with a deficient secretion of urine, especially if it be of a low specific gravity, and coagulable, and still more, if anasarcous swellings of the extremities have accompanied the headache, or it have succeeded to the rapid disappearance of such swellings, ought to be looked on with very great anxiety, and be met by immediate and energetic treatment. Headache, of a more or less severe and continued character, is the almost invariable precursor of this form of hydrocephalus; and an acquaintance, not very limited, with this, as well as other forms of dropsical effusion succeeding to scarlatina, has convinced me, that, whenever it occurs in the circumstances I have mentioned, and is attended by a loaded or furred tongue, and any degree of febrile action, we are warranted in regarding it as indicative of such congestion within the head as will, if not removed, be, in all probability, speedily followed by convulsions and coma.

"It should ever be recollected that it is not the severe forms of scarlatina that are most frequently followed by this important and dangerous sequela, but the milder forms, in which the rash has been trifling, and that have been attended by little angina, and no affection of the glands. Some exposure to cold will almost always be found to have been the exciting cause of the affection; after which the children suddenly lose their appetite, and complain of more or less languor, and frequently of pains in different parts of the body, and the urine becomes scanty and altered in quality. These symptoms of general derangement of the system are more marked in some cases than in others, and convulsions, or rapid coma, may come on *apparently* without any precursory symptoms, or without headache; and, therefore, to a certain extent, the usual statement may be correct, that the form of cerebral affection we are alluding to, is often very insidious. But my experience would rather lead me to say, that it was chiefly characterised by the very rapid manner in which it runs its course, when once developed; and that the preliminary symptoms referred to, particularly the headache, have always been present, though unnoticed or disregarded.

It has certainly not occurred to me to meet with any difficulty in detecting either the precursory symptoms of this form of disease, or in recognising its true nature in the subsequent stages." 164.

The subacute, or nervous variety of hydrocephalus is often of difficult detection, and then it becomes a matter of great nicety to decide whether the cephalic symptoms result from primary affection of the brain, or are merely secondary to, or sympathetic of, other disorders of the system. Every practitioner is aware of the great difficulty in this way experienced as regards infantile remittent fever. The following remarks, on the distinction between these diseases are worthy of attention.

" Dr. C. Smyth, who has, in many respects treated of the diagnosis of the simple forms of hydrocephalus more satisfactorily than most other writers, lays particular stress upon the admixture and alternations of the symptoms of irritation and those of oppression as distinctive of hydrocephalus. The association of these two classes of symptoms is remarkable, and can hardly fail to be noticed. Extreme irritability of the stomach, denoted by nausea and frequent vomiting, is in contrast with the remarkable insensibility of the bowels, as indicated by obstinate constipation. Morbid sensitiveness to light, and sound, and touch, are in opposition to the sluggishness of the pupil and constant drowsiness; whilst the slow breathing and sighing often contrast as forcibly with the quick pulse. Does any other disease present such an association of symptoms? 'I am perfectly aware,' says Dr. Smyth, 'and readily allow, that many of the symptoms of irritation are caused equally by teeth, worms, or other irritating matters, affecting the alimentary canal; but I do affirm that no irritation causes the constant combination of opposite symptoms above described.' " 167.

Another characteristic of the disease, of scarcely less importance, Dr. Bennett says, is the remarkable fluctuations and variability of the symptoms. "The children are often in a state of deep coma one day, and the next easily roused and perfectly conscious. The vomiting at one time is perpetually occurring, and at another the stomach shews no evidence of morbid irritability; the limbs are now convulsed, and now paralysed; the pulse for a few hours quick, then falling to the natural standard: now regular, now irregular and intermitting: the face flushed one moment, and pale the next, and so of all the more important functions."

The author next considers the diagnostic value of the various individual symptoms. *Headache* appears early and is seldom absent except in very chronic cases, while, in the hydrocephaloid affections, it is often not present. *Stupor* is always present in the true disease, as well as in the pseudo affections, and, therefore, its absence is of great negative value in determining that serious cerebral disease does not exist. The apparent stupor, in many febrile affections, is distinguished, by the ease with which the child may be roused, and the ready manner in which the pupil will then act. *Vomiting* is almost always present, and is valuable in diagnosis, in proportion as it occurs spontaneously, or from mere change of posture. Obstinate *constipation*, an early symptom, becomes the more observable when, as it often does, it succeeds to an undue relaxation of the bowels, which may have existed prior to the cerebral disease. This is not observed in the spurious affections. The green and gelatinous condition of the *stools* offers only some corroboration of other symptoms. The *morbid sensibility of the eye and ear* is found equally in the hydrocephaloid disease, but its non-occurrence, in the early stage, affords strong presumption of the non-existence of cerebral inflammation. Dr. Bennett lays little stress on the condition of the *pupil*, which, although usually contracted at the commencement of the sthenic variety, may assume any appearance in the others. Contrary to Dr. Green, and the French observers, he has found the *pulse* quick and irregular at the commencement of even the strumous and low varieties of the disease. But prognosis should not be founded too hastily upon the excessive rapidity or slowness of pulse, which sometimes precede the fatal termination, as this is often falsified. The state of the *respiration* is diagnostic. Simple increased rapidity is characteristic of fever, but irregular, sobbing, or suspirious breathing, betokens hydrocephalus. The importance of *convulsions*, and of their period of occurrence, as relates to diagnosis, has been exaggerated. One of the most valuable symptoms, considered by Smyth and Gölis as almost pathognomic, (of the scrofulous variety at least,) is a *retracted state of the abdomen*, which is produced by no other disease of children, and resembles the appearance induced by painter's colic in the adult.

### Chap. VII.—Treatment.

The acute and sthenic varieties, occurring in healthy children, must be met with the most energetic depletory treatment, and, restricted to such cases, the proceedings recommended by Clarke, are admirable. So, too, where the affection follows scarlatina, or other exanthemata, vigorous depletion and free purgation, will alone save the child—the skin being simultaneously excited by friction and diaphoretics.

All this is plain sailing enough, and no one will gainsay these doctrines, but the low, nervous, or strumous variety is that which truly offers difficulty, both in diagnosis and treatment; and we are disposed to value highly the present work, principally from the able protest the author enters against indiscriminate bleeding in these cases. Even in this variety, he bleeds at the very commencement, to relieve the headache, and place the brain in a condition more amenable to other remedies; but, he regards attempts to *cure* it by this means as a most fatal fallacy. As a general rule, leeches, or a few ounces of blood taken by cupping, from the vicinity of the mastoid process, will suffice, and, indeed, even this can only be employed at an early stage of the disease.

" I am quite aware that, in the eyes of many practitioners, of the present day, these very modified views respecting the employment and utility of bleeding will meet with but little favour. They have, however, been forced on me by my own observation and experience of the disease; and their soundness has, I think, been proved by the increased success that has attended their adoption. Let it not be forgotten, however, that our success will be proportioned to the judicious manner in which we combine other parts of the treatment with this modified employment of depletion. It appears to be an error, into which many have fallen, to suppose that if the treatment is to be antiphlogistic, and depletion resorted to at all, diuretics, sedatives, or any other remedies adopted to counteract the tendency to effusion, or other changes unconnected with inflammation, may be dispensed with. But it is far otherwise. The true secret of success consists, not merely in preventing or counteracting the attendant, and often accidental, inflammation, but also in allaying irritation of the nervous system, and in obviating the tendency to effusion and to exhaustion." 196.

The application of *cold* is valuable, when duly directed, but often hurtful when left to the nurse. The douche is confined to the active form of the disease, and, in all cases, even the application of a lotion must be guided by the state of the pulse and the temperature. Evaporation takes place more completely from the cropped hair than from the shaved head. *Purgatives* are valuable in all forms of the disease, and a full dose of calomel and purgative enemata should be given as early as possible, and, where vomiting resists every thing else, a drop of croton oil may be placed on sugar upon the tongue. Except when gastro-enteritis is present, a free action must be maintained throughout the duration of the disease. *Blisters,* delayed in the sthenic, are useful in the low form from the commencement, applied to the nape, thighs and calves, in which situations, too, sinapisms have their utility.

*Narcotics and Sedatives.*—The author believes these have been too sparingly employed. When after the active depletion, required for the active form of the disease, great irritation of the nervous system remains, *opium,* in discriminating doses, (the contractility of the pupil, as observed by Dr. Holland, must contra-indicate it) may be given with great advantage; and, even when the skin is dry, and the tongue and lips parched, Dover's powder has often the happiest effect. The more the case seems one of irritation the earlier must this means be resorted to; and, in the latter and apparently hopeless stage, although the coma may be encreasing, opiates have sometimes saved the child. Sedatives much assist the convalescence after severe depletory treatment; and they are of the greatest utility in cephalic irritation, or erethism of the brain, produced by causes acting at a distance—care being taken to remove these also, as far as possible. Where the mere allaying of irritation, and the procuring of sleep are required, the author prefers muriate of morphia, but, where the tonic properties of opium are also to be obtained, he gives laudanum or Dover's powder the preference. Hyoscyamus is often useful, but in a far inferior degree to opium. For a child of one year old one drop of laudanum is a sufficient dose, but if, in three or four hours, no unpleasant symptom has occurred, it may be encreased.

*Mercury.*—Dr. Bennett does not agree in the encomia which have been bestowed upon this drug, although it has sometimes proved beneficial in the latter stages. It is an useful addition to opiates, preventing constipation and obstruction of biliary secretion, while, calomel, given as a purgative, or combined with diuretics, is very valuable.

*Diuretics.*—The author considers these have been far too much neglected. The tincture of squill, combined with the spirit. ether. nit. and m. camph. is very useful in the latter stage of the low form, as is the combination of the powder with calomel and opium.

*Iodine* has frequently been found of decided advantage, and the hydriodate of potass has relieved cases, apparently sunk into a hopeless state.

*Tonics and Stimuli.*—These have not been sufficiently resorted to in this country in the latter stages, when they are often very useful; and it must be remembered also that the case may become aggravated by the too long persistence in an antiphlogistic diet.

*Preventive Treatment.*—Is much more likely to prove successful if the strumous pathology be admitted. This section contains some admirable remarks on the hygienic management of the predisposed, and on the avoidance and treatment of that prolific and unheeded source of disease—strumous dyspepsia.

An Appendix furnishes a few illustrative cases.

---

We have perused this work with unmixed satisfaction, and congratulate the London Medical Society at having elicited so valuable a contribution from its talented author. Much has been quoted, but we assure the reader that much important matter remains untouched; and, usually chary in recommending his purchasing books in this book-making age, we can conscientiously do so with respect to the present production. To the young practitioner, fresh from the schools, and imbued with the mere theoretical views too often taught therein, a work of this practical and discriminating character must prove of great value. To the more experienced it will afford a hope that a more just pathology may produce a more appropriate treatment, and will encourage them in the doubts and compunctions, they must have so often entertained, as to the soundness of the prevalent routine practice, recommended though it be by high-sounding names.

